# Hyperoxemia Is Associated With Mortality in Critically Ill Children

**DOI:** 10.3389/fmed.2021.675293

**Published:** 2021-06-07

**Authors:** Jonathan H. Pelletier, Sriram Ramgopal, Christopher M. Horvat

**Affiliations:** ^1^Division of Pediatric Critical Care Medicine, Department of Critical Care Medicine, UPMC Children's Hospital of Pittsburgh, Pittsburgh, PA, United States; ^2^Department of Pediatrics, Ann and Robert H. Lurie Children's Hospital of Chicago, Northwestern University Feinberg School of Medicine, Chicago, IL, United States; ^3^Division of Health Informatics, Department of Pediatrics, UPMC Children's Hospital of Pittsburgh, Pittsburgh, PA, United States

**Keywords:** oxygen, critically ill children, hyperoxaemia, mortality, review

## Abstract

Multiple studies among adults have suggested a non-linear relationship between arterial partial pressure of oxygen (PaO_2_) and clinical outcomes. Meta-analyses in this population suggest that high levels of supplemental oxygen resulting in hyperoxia are associated with mortality. This mini-review focuses on the non-neonatal pediatric literature examining the relationship between PaO_2_ and mortality. While only one pilot pediatric randomized-controlled trials exists, over the past decade, there have been at least eleven observational studies examining the relationship between PaO_2_ values and mortality in critically ill children. These analyses of mixed-case pediatric ICU populations have generally reported a parabolic (“u-shaped”) relationship between PaO_2_ and mortality, similar to that seen in the adult literature. However, the estimates of the point at which hyperoxemia becomes deleterious have varied widely (300–550 mmHg). Where attempted, this effect has been robust to analyses restricted to the first PaO_2_ value obtained, those obtained within 24 h of admission, anytime during admission, and the number of hyperoxemic blood gases over time. These findings have also been noted when using various methods of risk-adjustment (accounting for severity of illness scores or complex chronic conditions). Similar relationships were found in the majority of studies restricted to patients undergoing care after cardiac arrest. Taken together, the majority of the literature suggests that there is a robust parabolic relationship between PaO_2_ and risk-adjusted pediatric ICU mortality, but that the exact threshold at which hyperoxemia becomes deleterious is unclear, and likely beyond the typical target value for most clinical indications. Findings suggest that clinicians should remain judicious and thoughtful in the use of supplemental oxygen therapy in critically ill children.

## Introduction

Therapeutic oxygen administration has been studied since the early 1900s (1). Oxygen therapy has expanded dramatically in the last century. Today, many hospitalized patients and the majority of intensive care patients receive some form of supplemental oxygen (2, 3). Though the link between hyperoxia (supranormal oxygen delivery) and mortality was documented as early as the 1780s (4), there has been increasing interest in understanding this relationship in the past two decades. The association between hyperoxemia (supranormal blood oxygen content) and poor outcomes was first described among critically ill neonates in the 1990s (5, 6), culminating in recommendations to provide room air concentration oxygen to critically ill neonates in national guidelines (7). These findings prompted further research to evaluate the potential adverse effects of high oxygen concentration in other populations; initially adults, and more recently, among pediatric patients. In this review, we summarize recent findings with respect to the association between hyperoxemia and outcomes in critically ill pediatric patients. For an in-depth review of oxygen physiology in premature infants and in the delivery room, the reader is directed elsewhere (8–12).

## Mechanisms of Oxygen Toxicity

A number of reasons have been postulated to account for the potential toxicity of supranormal partial pressures of oxygen (4, 13). Mammalian cells depend upon mitochondria to generate adenosine triphosphate using aerobic metabolism (14). This process always generates small amounts of reactive oxygen species (ROS) (14). Normal intracellular signaling depends upon a balance of ROS generation and scavenging by enzymes such as superoxide dismutase (15, 16). When scavenging mechanisms are overwhelmed, ROS cause DNA damage, protein denaturation, and cell membrane damage (lipid peroxidation) (4, 15, 17, 18). High fractions of inspired oxygen (FiO_2_), particularly ≥0.6, have been found to cause alveolar mitochondria to generate an excess of reactive oxygen species, overwhelming normal scavenging mechanisms, and leading to cell death (4, 15, 17, 18). In addition to acting as direct cellular toxins, ROS also trigger proinflammatory signal cascades, neutrophil and macrophage recruitment, and platelet aggregation (4, 19).

Adults predominantly suffer acute lung injury secondary to hyperoxia (4, 19). By contrast, the premature and newborn population appears to be especially vulnerable to the multi-system effects of hyperoxia due to poorly developed ROS scavenging mechanisms (10). In this population, hyperoxia has been associated with retinopathy (20), intraventricular hemorrhage (10, 21), necrotizing enterocolitis (21), and cancers (particularly leukemia) (22, 23).

## Brief Review of Pivotal Adult Studies

Prior studies in adults have consistently suggested that an arterial partial pressure of oxygen (PaO_2_) ≥300 mmHg (hyperoxemia) is associated with poorer survival (24–26) and neurologic outcome (27) following cardiac arrest. One recent meta-analysis, summarizing results from 25 randomized controlled studies with >16,000 patients having a variety of critical illnesses suggested a liberal oxygen strategy was associated with a higher relative risk of mortality, both at 30-days and at longest follow up, supporting a conservative approach in the use of supplemental oxygen (28). However, only 2/25 of the original studies included in this analysis showed significant benefit associated with a conservative oxygen strategy (29, 30).

To date, one pilot study and two full randomized controlled trials have evaluated conservative vs. liberal oxygen therapy in mixed-diagnosis critically ill adult populations (30–32). These trials came to conflicting conclusions. A single-center, open-label trial randomized 434 critically ill mechanically ventilated adults to target PaO_2_ 70–100 mmHg and oxygen saturation (SpO_2_) 94–98% vs. PaO_2_ ≤150 mmHg and SpO_2_ ≥97% (30). This study showed a 42.6% reduction in all-cause ICU mortality (11.6 vs. 20.2% in the conservative vs. liberal groups, respectively, *p* = 0.01), as well as lower incidences of shock, liver failure, and bacteremia (30). However, this trial excluded patients with exacerbations of chronic obstructive pulmonary disease, and was terminated early due to an earthquake, resulting in a reduction of the sample size to 72.7% of the target (30). A pilot study examined SpO_2_ targets of 88–92% vs. ≥96% in 102 critically ill adults (31). They found non-significant decreases in 90 day mortality in the conservative oxygen therapy group, with a hazard ratio of 0.77 (95% CI, 0.40–1.50; *P* = 0.44), and a hazard ratio of 0.49 (95% CI, 0.20–1.17; *P* = 0.10) in patients with a PaO_2_/FiO_2_ ratio <300 (31). A subsequent full randomized controlled trial did not show benefit (32). This trial analyzed 965 critically ill adults randomized to receive “usual care” (where the lower SpO_2_ goal was set by the clinician, and there was no upper SpO_2_ goal) or “conservative oxygen therapy” (where the lower SpO_2_ goal was set by the clinician, but FiO_2_ was actively weaned for SpO_2_ ≥97%) with a primary outcome of number of ventilator free days by day 28 (32). This trial found no significant difference in ventilator free days, or the secondary outcomes of 90-day or 180-day mortality (32). However, only 138/965 (14.3%) of included patients had primary respiratory diagnoses, and the difference between mean FiO_2_ and PaO_2_ between the groups was very small (32). After the first day, both groups had mean FiO_2_ values ≤0.37 and mean PaO_2_ values ≤100 mmHg (32).

Taken together, these data suggest that extreme hyperoxemia is associated with worse outcomes, though the exact threshold for harm is uncertain. Similarly, it is unclear which has a greater impact: a high FiO_2_ or high PaO_2_. Regardless, and perhaps because of the conflicting evidence, clinicians are generally less attentive to hyperoxemia than to hypoxemia (33–35), causing patients to be exposed to higher FiO_2_ than needed.

## Review of Pediatric Literature

In the past decade, there have been 12 pediatric studies, encompassing a total of 26,838 pediatric ICU admissions, evaluating the association between PaO_2_ and mortality [Table 1; (36–47)]. While the results of individual studies vary, several studies have identified a second-order polynomial (“u-shaped”) relationship between PaO_2_ and mortality among critically ill children (36, 37, 41, 47). These studies can generally be grouped according to their included population.

**Table 1:** Summary of pediatric studies examining the relationship between PaO_2_ and mortality.

**References**	**Study type**	**Population**	**Number**	**Predictor**	**Outcome**	**Analysis**	**Key findings**
**All diagnoses**							
Raman et al. (36)	Retrospective, observational	Single-center PICU	7,410 admissions	Admission hyperoxemia (>300 mmHg) and hypoxia (<60 mmHg)	In-hospital mortality	Polynomial regression with modified PIM-2 risk-adjustment, age and gender	U-shaped association between PaO_2_ and mortality Modified standardized mortality ratio 1.34 for hypoxemia, and 0.83 for hyperoxemia
Numa et al. (37)	Retrospective, observational	Single-center PICU	1,447 admissions	Admission PaO_2_ in 50 mmHg bands	In-hospital mortality	Polynomial regression with modified PIM-3 based risk-adjustment	U-shaped mortality curve; with l mortality in 101–200 mm Hg bands; higher mortality in <50 mmHg and >350 mmHg bands aOR for mortality of 2.66 in hyperoxemic (250 mmHg) group
Peters et al. (38)	Pilot open randomized-controlled trial	Multi-center (3 sites) PICU	117 admissions	SpO_2_ >94% vs. SpO_2_ 88–92%	N/A (pilot study)	Descriptive statistics	No difference in ICU mortality, ventilator free days at day 30, or ICU length of stay
Ramgopal et al. (39)	Retrospective, observational	Single-center PICU	6,250 admissions	Hyperoxemia (≥300 mmHg) at any time	In-hospital mortality	Logistic regression using adjusted PELOD, use of ECMO, number of ABG collected	aOR for hyperoxemia 1.78 (95% CI 1.36–2.33); odds of mortality rising with number of hyperoxemic ABG
Ramgopal et al. (40)	Retrospective, observational	Single-center PICU	4,093 admissions	Hyperoxemia (≥300 mmHg) and “extreme hyperoxemia” (≥550 mmHg) −6 to +6 h of PICU admission	In-hospital mortality	Logistic regression using adjusted PRISM-IV	Hyperoxemia not associated with increased adjusted mortality risk (aOR 1.38, 95% CI 0.98–1.93). “Extreme hyperoxemia” associated with higher aOR of mortality (aOR 2.44, 95% CI 1.32–4.50)
Pelletier et al. (41)	Retrospective, observational	Multi-center (6 sites) PICU	4,469 admissions requiring mechanical ventilation	Maximum PaO_2_ in 100 mmHg bands	In-hospital mortality	Polynomial regression, adjusted for demographic variables and clinical variables	Maximum PaO_2_ associated with mortality (adjusted standardized mortality ratio 1.27, 95% CI 1.23–1.32)
**Post-cardiac arrest**							
Ferguson et al. (42)	Retrospective, observational	Multicenter (33 sites), post-cardiac arrest	1,875 post-arrest patients	Hypoxia (<60 mmHg) and Hyperoxemia (≥300 mmHg) collected after PICU physician evaluation to 1 h after PICU admission	Mortality in PICU	Polynomial regression, adjusting for demographics, site of arrest, congenital cardiac disease, supportive therapies, and PIM-2	Hyperoxia found in 24% of initial ABG Hyperoxemia associated with mortality (aOR 1.25; 95% CI 1.17–1.37)
Del Castillo et al. (43)	Prospective, observational	Multicenter, post-cardiac arrest	223, post-arrest patients	Hypoxia (<60 mmHg or PaO_2_/FiO_2_ <200) and Hyperoxemia (≥300 mmHg or PaO_2_/FiO_2_ >300) from first ABG after admission	In-hospital mortality	Chi-squared test	Mortality with hyperoxia 33%, hypoxemia 29.8%, and normoxemia 35.8%; not statistically significant (*p* = 0.16)
Guerra-Wallace et al. (44)	Retrospective, observational	Single center, post-cardiac arrest	74 post-arrest patients	Any hyperoxemia (>300 mmHg)	6-month mortality	Descriptive	38 patients with at least one PaO_2_ >300 mmHg; no association with 6-month mortality among patients with hypoxemia or hyperoxemia
van Zellem et al. (45)	Retrospective, observational	Single center, post-cardiac arrest; receiving mild therapeutic hypothermia	200 post-arrest patients	>250 mmHg any time following arrest; derived from cutoff analysis	In-hospital mortality	Logistic regression adjusted for age, gender, type of resuscitation, location, rhythm, pH, lactate Cumulative PaO_2_ analysis using AUC determined by trapezoidal method	Univariable analysis suggested improved survival odds with hyperoxemia (not found after adjustment of confounders) No association between duration of cumulative PaO_2_ AUC and mortality
**Traumatic brain injury**							
Ramaiah et al. (46)	Retrospective, observational	Single center, post-TBI	194 admissions	Hypoxemia (<60 mmHg) Hyperoxemia (300–500 mmHg)	In-hospital mortality	Logistic regression adjusted for age, injury severity score	Hyperoxemia associated with higher odds to survival (aOR 8.02, 95% CI 1.73–37.10)
**Extracorporeal membrane oxygenation**							
Cashen et al. (47)	Retrospective, observational	Multi center (8 sites), within 48 h of ECMO cannulation	484 admissions	Hyperoxemia (>200 mmHg)	In-hospital mortality	Logistic regression, adjusted for carbon dioxide level, lactate, diagnosis, and pre-term status	Lowest mortality in group with PaO_2_ 60–99 mmHg, stepwise increases above 100 mmHg Hyperoxemia associated with increased odds of in-hospital mortality (OR 1.03 95% CI 1.01–1.04 for every 10 mmHg)

*ABG, arterial blood gas; ECMO, extracorporeal membrane oxygenation; N/A, not applicable; PICU, pediatric intensive care unit; PIM, pediatric index of mortality; PRISM, pediatric risk of mortality*.

### Pediatric ICU Patients With Heterogenous Diagnoses

Six of the twelve studies considered heterogenous patients admitted to the pediatric ICU (36–41). Of these, four demonstrated increased patient mortality with PaO_2_ values above 300–400 mmHg, and one found this relationship only above 550 mmHg. Peters et al. conducted a pilot open randomized-controlled trial including 119 patients >38 weeks gestation and <16 years of age undergoing non-invasive or invasive mechanical ventilation (38). They randomized patients to target SpO_2_ >94% (liberal oxygen strategy) or 88–92% (conservative oxygen strategy). As it was a pilot study, there was no power calculation performed for clinical outcomes, which were available for 107/119 patients. There was no significant difference in ICU mortality (4/53 vs. 4/54 liberal vs. conservative, respectively), ventilator-free days, or ICU length of stay (38).

Numa et al. performed a single-center retrospective database analysis of arterial blood gas values obtained within 1 h of ICU admission (37). They demonstrated a polynomial relationship between PaO_2_ and mortality both before and after adjustment for Pediatric Index of Mortality-3 scores: patients with a PaO_2_ of 301–350 had standardized mortality ratio of 1.18 compared to those with PaO_2_ 251–300_._ When incorporated in a model including other variables associated with mortality, patients with PaO_2_ ≥250 mmHg had an adjusted odds ratio (aOR) of mortality of 2.66. In a similar investigation, Raman et al. performed a single-center retrospective analysis of the relationship between PaO_2_ on pediatric ICU admission and mortality before and after adjustment for Pediatric Index of Mortality-2 scores (modified to remove the PaO_2_/FiO_2_ ratio) (36). They included all patients admitted to pediatric, neonatal, or cardiac intensive care units. They found that both unadjusted and adjusted mortality were lowest in patients with PaO_2_ values between 187 and 300 mmHg.

Ramgopal et al. conducted a single-center retrospective analysis of PaO_2_ values at any point during ICU admission, and found that a maximum PaO_2_ ≥300 mmHg was associated with increased mortality after adjustment for a modified Pediatric Logistic Organ Dysfunction-2 score (aOR 1.75 95% confidence interval [CI] 1.33–2.29) (39). This study also suggested an exposure gradient: patients who had multiple PaO_2_ values ≥300 mmHg had higher risk-adjusted mortality than those with only a single value in this range (odds ratio for one PaO_2_ value ≥300 mmHg 1.47 95% CI 1.05–2.08 compared to odds ratio for at least three PaO_2_ values ≥300 mmHg 2.53 95% CI 1.62–3.94). In a second study, Ramgopal et al. also examined PaO_2_ values in the 12 h surrounding PICU admission when adjusting for a modified Pediatric Risk of Mortality IV score (40). While this study found that PaO_2_ ≥300 mmHg was not significantly associated with risk-adjusted mortality (OR 1.38 95% CI 0.98–1.93), a PaO_2_ ≥550 mmHg did reach the threshold of statistical significance (OR 2.44 95% CI 1.32–4.50) (40). Given that the point estimate of this latter study was still suggestive of a relationship between PaO_2_ ≥300 mmHg and risk-adjusted mortality, it is worth noting that a smaller sample size may have accounted for some of these differences: while the former study included 23,719 patients, the latter included a subset of fewer than 20% of those cases (39, 40).

Pelletier et al. performed a multicenter, mixed-diagnosis pediatric study examining the potential relationship between hyperoxemia and outcome (41). The authors performed a retrospective analysis of a federated electronic health record database from six large academic pediatric centers over 3 years (48, 49). Because the variables for previously-validated risk-adjustment models were not available for their cohort, they trained and validated a novel risk-adjustment model based on laboratory values, inotrope and vasopressor use, and demographic characteristics (c-statistic 0.86 [95% CI 0.79–0.92], area under the precision recall curve 0.39, standardized mortality ratio 0.96 [95% CI 0.75–1.23]), then added PaO_2_ to the model to determine the effect (41). They found that maximum PaO_2_ values above 384 mmHg within the first 72 h of ICU admission in mechanically ventilated children were associated with increased risk-adjusted mortality (41). This finding was stable in sensitivity analyses excluding children with chronic complex cardiovascular conditions (50), those with traumatic brain injury and carbon monoxide poisoning, and children presenting after cardiac arrest (41). The findings were also stable in a sensitivity analysis examining PaO_2_ values at any time during admission (41). However, when the cohort was narrowed to examine only PaO_2_ values within 24 h of admission, a higher threshold of hyperoxemia (≥486 mmHg) was associated with risk-adjusted mortality.

### Pediatric ICU Patients Admitted After Cardiac Arrest

There are currently four studies evaluating the relationship between PaO_2_ ≥300 mmHg and mortality in pediatric ICU patients admitted after cardiac arrest. Of these, one identified a significant relationship between hyperoxemia and mortality after adjustment for confounders, and the other three did not (42–45). Ferguson et al. first performed a retrospective observational multicenter database study of 1,875 children ≤16 years old admitted to a pediatric ICU following cardiac arrest (42). They found that PaO_2_ on the first blood gas obtained within 1 h of arrival to the pediatric ICU had a non-linear association with mortality. After adjustment for age, sex, arrest location, and Pediatric Index of Mortality-2 scores, they noted that the optimal first PaO_2_ was 60–75 mmHg, and that mortality rose both above and below this value. At the extremes, a PaO_2_ of 23 mmHg was associated with an increased aOR of mortality of 1.92 (95% CI 1.80–2.21), and a PaO_2_ value of 600 mmHg was associated with an increased aOR of mortality (1.25; 95% CI 1.17–1.37).

Del Castillo et al. performed a retrospective multicenter study of 223 patients aged 1 month to 18 years admitted after cardiac arrest in which they evaluated the relationship between arterial blood gas values immediately post-resuscitation and 24 h later with mortality. They found increased mortality in patients with PaO_2_ ≥300 mmHg (52.6%) vs. those with PaO_2_ <60 mmHg (42.4%) or those with PaO_2_ values ≥60 mmHg and <300 mmHg (40.7%), but the difference was not statistically significant (*p* = 0.61).

Guerra-Wallace et al. conducted a single-center retrospective chart review of 74 patients admitted to a pediatric ICU after cardiac arrest (44). Their study was notable for including the highest and lowest PaO_2_ of patients within the first 24 h after admission, and for excluding patients who died within 48 h of admission. They found no association between PaO_2_ values <60 mmHg or ≥300 mmHg and unadjusted mortality at 6 months (44). van Zellem et al. conducted a single-center retrospective study of patients >28 days to <18 years old admitted to a pediatric ICU after cardiac arrest (45). They did not find a significant association between PaO_2_ values ≥300 mmHg and mortality before or after adjustment for confounders. However, in the subset of patients concomitantly undergoing moderate therapeutic hypothermia they found an association between higher cumulative PaO_2_ values in the first 24 h and survival (45).

### Pediatric ICU Patients Admitted With Severe Traumatic Brain Injury

A single study by Ramaiah et al. retrospectively evaluated the association between PaO_2_ values and mortality in patients age ≤14 years admitted to a single pediatric ICU (46). In contrast to the majority of other studies, they found that admission PaO_2_ 300–500 mmHg was associated with increased survival to discharge (aOR 8.02 95% CI 1.73–37.10) (46). While not included in their multivariable model, the authors found that PaO_2_ values <100 mmHg or >500 mmHg were associated with worse survival (46). In addition to being notable as the only study to find a favorable association between PaO_2_ values ≥300 mmHg and survival to discharge, this study is also notable for its effect size (46). No other study including <1,000 admissions found any significant effect of hyperoxemia, albeit in different populations (46). While the authors did not analyze collinearity between PaO_2_ values and chest trauma in this cohort, significantly fewer survivors than non-survivors had concomitant chest trauma [39/162 [24.1%] vs. 13/32 [40.6%], *p* = 0.05; (46)]. In a separate retrospective review of traumatic brain injury, Michaud et al. secondarily found that PaO_2_ and chest trauma were collinear in their population, and that hypoxemia and chest trauma were associated with poor outcome in traumatic brain injury (51). Thus, it remains unclear whether higher PaO_2_ values are beneficial in traumatic brain injury, or whether these values are confounded by polytrauma.

### Pediatric ICU Patients on Extracorporeal Membrane Oxygenation

Cashen et al. conducted a multicenter, retrospective database study of 484 patients age <19 years treated with extracorporeal membrane oxygenation (ECMO) for heterogenous diagnoses (47). They analyzed arterial blood gas data obtained within 48 h of ECMO cannulation. They defined hyperoxia as PaO_2_ >200 mmHg. They found that patients with a maximal PaO_2_ 60–99 mmHg had the lowest mortality. Mortality was higher for patients with a maximal PaO_2_ <60 mmHg, and there were stepwise increases in mortality for every 100 mmHg increase above 100 mmHg. Patients with a maximum PaO_2_ >200 mmHg had substantially higher in-hospital mortality than those that did not (167/331 [50.5%] vs. 48/153 [31.4%]), respectively. In multivariable logistic regression accounting for lactate, diagnosis, and age, every 10 mmHg increase in PaO_2_ was associated with an increased odds of mortality (OR 1.03 95% CI 1.01–1.04). However, the hyperoxia group was notable in that it had a significantly higher proportions of patients with a cardiac indication for ECMO (48.9 vs. 24.8%), ECPR (16.0 vs. 10.5%), and venoarterial cannulation (92.1 vs. 75.2%).

### Areas of Controversy in the Pediatric Literature

Existing literature examining the relationship between hyperoxemia and outcomes among critically ill children is limited and predominantly observational. Additionally, the threshold at which hyperoxemia may result in harm is unclear. Several pediatric studies support the idea of harm beginning at ~300 mmHg, but no study <1,000 patients has demonstrated harm (43–46), and one study only showed a significant association between PaO_2_ ≥550 mmHg and mortality (40). It is also interesting to consider whether PaO_2_ is the right metric for oxygen toxicity at all; because excess oxygen is known to act as a local pulmonary mitochondrial toxin (4, 17, 18) as well as a systemic inflammatory mediator (4, 19), it may well be that the relationship between FiO_2_ and mortality is much more important than the relationship between PaO_2_ and mortality.

Additionally, given their retrospective nature, all of the current pediatric studies are vulnerable to confounding by indication. For example, it may be that patients with high PaO_2_ values merely represent more gravely ill patients treated with high FiO_2_ as part of resuscitation bundles. As such, hyperoxemia may be associated with illness severity but not causative of clinically meaningful organ dysfunction. Expert opinion has queried whether hyperoxemia may be a spurious marker of death by neurological criteria as apnea testing is conventionally performed with an FiO_2_ 1.0 or evaluation for organ transplantation in moribund patients (36). However, several of the above studies support an association that would not be influenced by assessments for neurological death. Two studies evaluated PaO_2_ values within the first 6–24 h after admission (37, 39), and several evaluated PaO_2_ values either immediately on admission, or within 1 h (34, 45, 46). All of these studies found that hyperoxemia was associated with an increase in mortality, albeit at different threshold PaO_2_ values.

Given that there is likely to be little clinical benefit to prolonged periods of high FiO_2_ exposure, and given the array of preclinical and clinical studies suggestive of possible harm (4, 14–21, 52–56), this question is unlikely to be answered directly in pediatric randomized controlled trials or prospective studies. It is difficult to imagine that there is clinical equipoise to randomize children to a target PaO_2_ of ≥ 300 mmHg, except perhaps in the specific situation of severe traumatic brain injury (43). Larger trials evaluating conservative oxygen administration strategies and targets compared to usual care are more likely to receive support. Recognizing the potential harm caused by hyperoxemia, the American Heart Association advocates a target oxygen saturation of 94–99% in the post-resuscitation setting (48, 57) (p4). As learning health systems evolve to become more efficient with patient identification, clinical trial enrollment and data collection, there may be an opportunity to study oxygen delivery strategies among critically ill children with a prospective, randomized design that would otherwise have been deemed infeasible due to costs.

## Conclusion

Of the twelve pediatric studies evaluating the relationship between PaO_2_ and mortality, seven found a potentially harmful association between hyperoxemia and mortality. Notably, the five studies that did not find such an effect had substantially smaller sample sizes. The relationship between PaO_2_ values and mortality in the pediatric ICU setting appears to be polynomial (“u-shaped”), with both hypoxemia and hyperoxemia associated with increased mortality. Several studies demonstrated that this association persisted after adjustment for demographics and severity of illness. The exact value at which hyperoxemia is potentially harmful is unclear but is likely between 250 and 400 mmHg. These studies reinforce preclinical and healthy volunteer studies that demonstrate a potentially deleterious effect of excess oxygen therapy. The single exception to this trend is in traumatic brain injury patients, where a single small study demonstrated a favorable association between PaO_2_ values 300–500 mmHg and mortality. It remains unclear whether hyperoxemia causes clinical harm, or is a marker of illness severity. However, in the absence of a specific indication, pediatric providers should be prudent with the use of oxygen therapy and avoid PaO_2_ values ≥300.

## Author Contributions

JP wrote the first draft of the manuscript. SR wrote the first draft of the table. All authors critically revised the manuscript. All authors have reviewed the final version, agreed upon submission, and agree to be accountable for the content of the work.

## Conflict of Interest

The authors declare that the research was conducted in the absence of any commercial or financial relationships that could be construed as a potential conflict of interest.
